# A case of bronchiolitis obliterans in a young child

**DOI:** 10.4103/0972-5229.76086

**Published:** 2010

**Authors:** Vaibhav Keskar, Milind S. Tullu, Sandeep B. Bavdekar

**Affiliations:** **From:** Department of Pediatrics, Seth G.S. Medical College and KEM Hospital, Mumbai, India

**Keywords:** Bronchiolitis obliterans, chest, child, lung, mechanical ventilation, pneumonia, steroids

## Abstract

Bronchiolitis obliterans (BO) is an infrequent chronic obstructive pulmonary disease that follows an insult to lower respiratory tract. BO following a severe lung infectious disease is the most common form reported in children. It implies a chronic necrotizing and ultimately fibrosing process affecting the small airways which results in progressive obliteration with resultant obstructive lung disease. The characteristic symptom-complex includes dyspnea, chronic cough, sputum production and wheezing. Although histopathology remains the gold standard for diagnosis, computed tomographic scan is of great help in diagnosis. Etiological role of mechanical ventilation has not yet been studied well. We report a case of BO in a three year old child occurring after an episode of lower respiratory tract infection requiring prolonged mechanical ventilation with subsequent development of ventilator-associated pneumonia. Our patient had a protracted clinical course with good response to corticosteroids.

## Introduction

Bronchiolitis obliterans (BO) is an infrequent chronic disorder characterized by (partial or complete) obstruction of bronchi and bronchioles by fibrous tissue following an insult to the lower respiratory tract.[1] In children, BO is frequently preceded by respiratory tract infection by adenovirus, influenza, or measles. It also occurs after lung transplant.[2–6] Reported sequelae include chronic atelectasis, bronchiectasis, and unilateral hyperlucent lung syndrome (Swyer-James syndrome).[4,7] Murtagh P *et al*, have recently shown that the sequelae in 415 patients with acute lower respiratory infection (due to adenovirus infection) included bronchiectasis and chronic atelectasis, but no cases of hyperlucent lung syndrome.[8] We describe a case of BO and discuss the possible causative role of mechanical ventilation.

## Case Report

A three-year-old boy presented with history of cough, cold and fever for seven days, sleepiness for four days and multiple generalized tonic-clonic convulsions for 1 day. History of such episodes, prior hospitalization, tuberculosis or tuberculous contact was absent. On examination, he had heart rate of 130/min, respiratory rate of 54/min with severe respiratory distress and normal blood pressure. Bronchial breath sounds and crepitations were heard in right supra-mammary, mammary, infra-mammary and axillary areas with decreased breath sounds in left infra-axillary area. The central nervous system examination revealed stuporous sensorium and normal cranial nerve examination. He had hypotonia, depressed deep tendon reflexes and extensor plantar reflexes.

**Figure 1:**
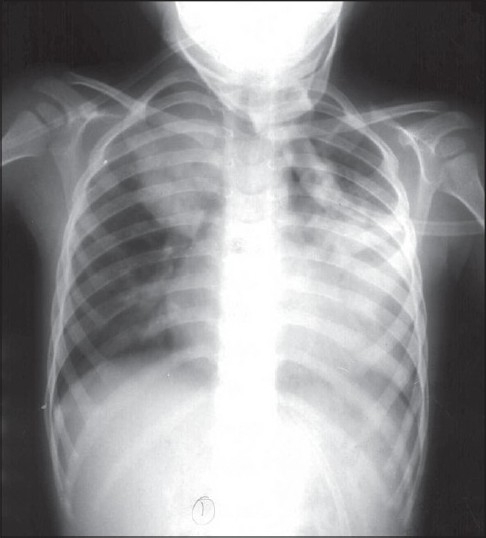
Chest radiograph on admission showing right upper and middle zone pneumonia along with pleural effusion on left side.

**Figure 2:**
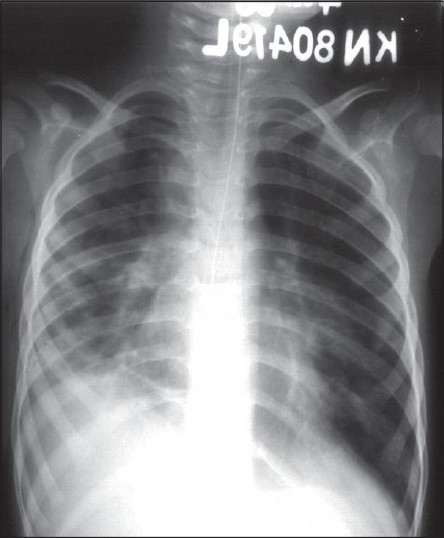
Radiograph showing ventilator-associated pneumonia in right lower zone.

**Figure 3:**
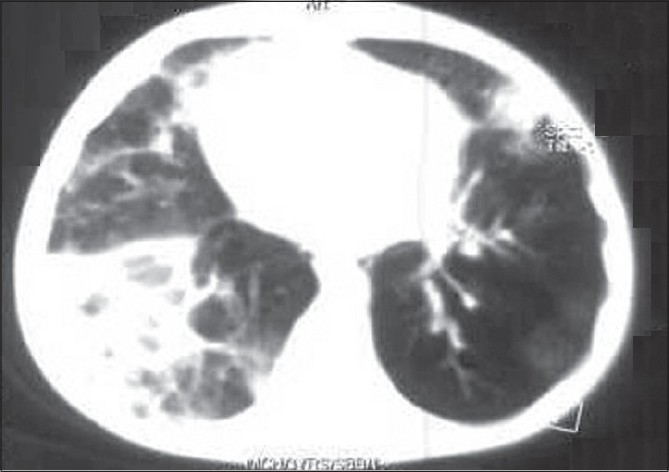
CT chest at the end of 3 weeks of illness showing multiple areas of consolidation in both the lung fields with ground glass opacities and mediastinal lymphadenopathy.

The hemogram showed hemoglobin concentration of 10.8 g%, leukocyte count of 7800/μl with polymorphonuclear predominance and platelet count of 1,20,000/μl. Serum electrolytes and creatinine were normal. Liver transaminases were moderately elevated (ALT 347 and AST 108 IU/L, respectively). Cerebrospinal fluid (CSF) analysis showed protein and sugar concentrations of 28 mg/dl and 66 mg/dl, respectively; with 2 lymphocytes and 15 RBCs on smear. CSF culture did not grow any organism. Mantoux test was non-reactive. Gastric aspirate was negative for acid fast bacilli. Blood and tracheal aspirate culture were negative. Chest radiograph showed right upper and middle zone pneumonia along with pleural effusion on the left side [Figure 1], which was confirmed on ultrasonography. We could not perform the IFI test for detecting adenovirus. Computed tomographic (CT) scan of brain was normal. Pleural fluid appearance was hemorrhagic with protein concentration of 2.2 g% and 350 white cells per μL (90% lymphocytes, RBCs of 0.19 million). Smear examination and culture of CSF did not show presence of any organism. He was treated with anticonvulsants (phenytoin and phenobarbitone followed by midazolam infusion for repeated convulsions) and intravenous antibiotics (cefotaxime). He also required mechanical ventilation for his respiratory failure. In view of hemorrhagic pleural effusion and non-response to antibiotics, anti-tuberculous drug therapy was initiated. New infiltrates were seen on chest radiograph in right lower zone after eight days of mechanical ventilation (ventilator-associated pneumonia) [Figure 2] and were treated with inj. vancomycin and meropenem. This treatment resulted in partial clinical response. He was ventilated for 10 days (maximum pressures used included a peak inspiratory pressure of 20cm in controlled mode and 16 cm in pressure-controlled synchronous intermittent mandatory ventilation- SIMV mode). After extubation, he was put on oxygen by mask (FiO_2_ = 0.4). In view of hypoxemia (PaO_2_ = 27 mmHg; SaO_2_ = 54%), the FiO_2_ was increased with the help of non-rebreathing mask. CT scan of chest done at the end of 3 weeks showed multiple areas of consolidation in both the lung fields with ground glass opacities and mediastinal lymphadenopathy [Figure 3]. The patient showed gradual improvement in sensorium and complete control of convulsions. Gradually, the oxygen requirement could be reduced; but hypoxia persisted (room air PaO_2_ = 34; SaO_2_ = 67%) and he continued to have bilateral lung crepitations.

**Figure 4:**
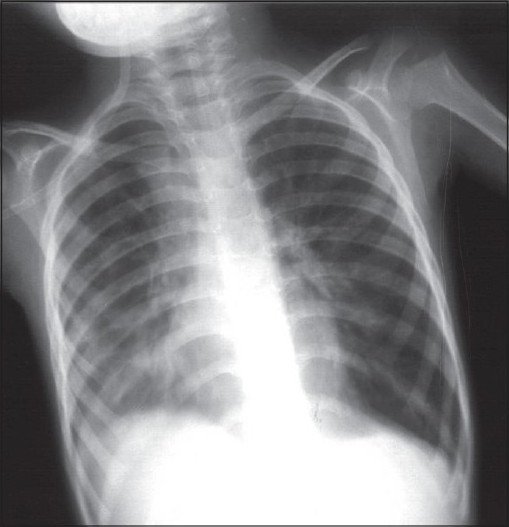
Chest radiograph at the end of two months of illness depicting hyperinflation.

**Figure 5:**
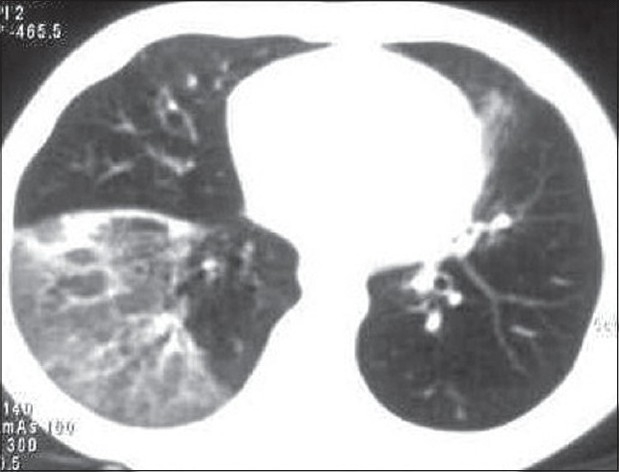
CT chest at the end of 10 weeks showing partially resolved pneumonia bilaterally and features of bronchiolitis obliterans (terminal bronchiolar dilatation with areas of air trapping).

**Figure 6:**
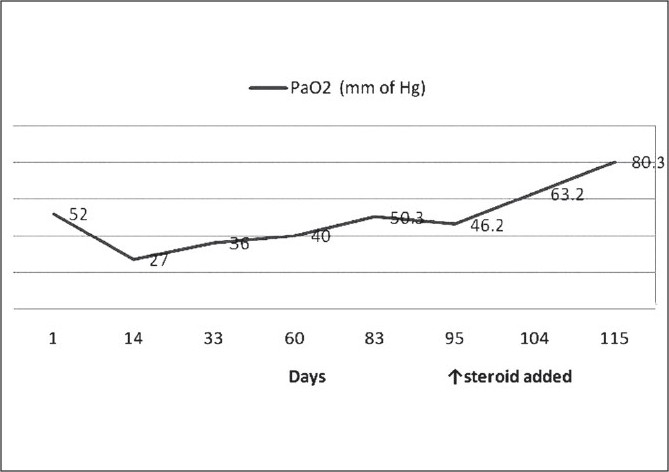
Serial PaO_2_ values.

At 10 weeks of hospitalization, in view of the clinical picture (chronic symptoms of cough and dyspnea), persistent desaturation at room air, radiological features (hyperinflation, Figure 4) and tomographic features (partially resolved consolidation and terminal bronchiolar dilatation with areas of air trapping; Figure 5), a diagnosis of BO was considered. He was treated with inhaled bronchodilators and therapy with oral prednisolone (1 mg/kg/day) was initiated. This was followed by gradual increase in PaO_2_ and at the end of 21 days of steroid therapy, he was maintaining PaO_2_ on room air [Table 1 and Figure 6]. He could be discharged after a 16-week hospital stay. At eight months follow-up, he has bilateral basal crepitations, normal PaO_2_ on room air, normal chest radiograph and no neurological deficits. He is presently on tapering doses of oral prednisolone.

**Table 1:** Serial blood gas values

Day	Clinical condition	PH	PaCO_2_	PaO_2_	O_2_ sat (%)	HCO_3_	Comment
1	Pre-intubation	7.45	38	52	88	27.2	Mechanically ventilated
2	Post-extubation	7.57	25	170	99.7	24	----
8	On weaning mode (CPAP)	7.5	33	108	98	25.8	Extubation planned
9	Post-extubation	7.45	39	203	100	27	FiO_2_ = 0.4
14	On room air	7.44	49.4	27	54	27.1	Cyanosis noted if kept off O_2_
20	On O_2_ by mask	7.38	50	122	99	29.6	------
25	On O_2_ by mask	7.53	40.6	64	95	33	O_2_ continued
33	On room air	7.42	42	36	73	27.9	O_2_ continued
44	On room air	7.42	46	34	67	24	O_2_ continued
60	On room air	7.37	29.4	40	74	17.5	O_2_ continued
70	On room air	7.46	36.5	48	86.4	25.7	O_2_ continued
83	On room air	7.5	31.4	50.3	89.4	24.5	Oral steroid started
95	On room air	7.4	36	46.2	82	22.4	-----
104	On room air	7.43	30.2	63.2	92.3	19.7	-----
115	On room air	7.36	29.3	80.3	94.9	16.3	Discharged

O_2_ = Oxygen; CPAP = Continuous positive airway pressure; PaO_2_ and PaCO_2_ = Arterial oxygen and carbondioxide concentrations; O_2_ sat = oxygen saturation; HCO_3_ = Bicarbonate.

## Discussion

BO implies a chronic necrotizing and ultimately fibrosing process affecting the small airways which results in progressive obliteration with resultant obstructive lung disease.[8] Pathologically, BO is characterized by bronchiectasis of the large airways and luminal obstruction with inflammation, granulation tissue, and/or fibrosis and obliteration of the small airways.[1] Known etiologies include infection (particularly adenovirus and Mycoplasma pneumoniae; also measles, Legionella, pertusis, influenza),[2–5] aspiration and toxic inhalation. Other cases have had predisposing factors such as Stevens-Johnson syndrome,[9] lung and bone marrow transplantation,[6] and connective-tissue disorders. In many cases, the etiology remains unknown.

Though BO has been seen to be associated in patients receiving mechanical ventilation for pulmonary conditions, it is still difficult to attribute an etiological role to mechanical ventilation.[10] Some feel that it may merely reflect the severity of the underlying initial insult to the lungs.[10] Our patient was ventilated for pneumonia for 10 days and later developed BO. It is possible that the initial injury to the lungs was exacerbated due to the mechanical ventilation. Murtagh *et al*, evaluated risk factors for BO and death.[8] They have shown that mechanical assistance was associated to BO in univariate analysis, but in the multivariate model mechanical assistance was no longer related to BO.[8] More than 30 days of hospitalization, multifocal pneumonia as initial clinical presentation and hypercapnia were risks factor related to BO in the population (117 patients of BO occurring post- adenovirus infection out of 415 patients with adenovirus infection).[8]

Very few studies have been done in context of BO in children.[4,7,10] Colom *et al*,[10] noted that adenovirus infection and mechanical ventilation were significant risk factors- 34% of patients with post-infectious BO required mechanical ventilation compared with only 3% of controls. In a case series of 19 children with post-infectious BO, Hardy *et al*,[4] observed that 12 patients were ventilated in the course of initial illness.

Unlike in case of infectious agents like adenovirus and mycoplasma, the causative role of mechanical ventilation has not been proved, although its association with BO has been noted.[10] While the fact that these children who later develop BO have history of mechanical ventilation, it may just reflect the severity of the initial insult. The role, if any, of volutrauma, atelectotrauma, barotrauma and biotrauma remain to be explored.[10] The use of corticosteroid therapy in the early phase has been shown to be beneficial in treatment of BO.[7] The benefit of corticosteroids use, both in acute and in chronic periods of the disease seems conflicting.[4,5,7] Though our case report does not confirm the role of mechanical ventilation in causing BO, further research into the risk factors for BO including the role of mechanical ventilation is warranted. This case is being presented for its rarity and to suggest possibility of mechanical ventilation as a causative factor in the pathogenesis of BO.
